# Equilibrium modeling of selenium binding from aqueous solutions by *Candida utilis* ATCC 9950 yeasts

**DOI:** 10.1007/s13205-018-1415-8

**Published:** 2018-08-27

**Authors:** Marek Kieliszek, Stanisław Błażejak, Kamil Piwowarek, Katarzyna Brzezicka

**Affiliations:** Faculty of Food Sciences, Department of Biotechnology, Microbiology and Food Evaluation, Warsaw University of Life Sciences−SGGW, Nowoursynowska 159 C, 02-776 Warsaw, Poland

**Keywords:** Selenium, Yeast, Kinetics, Isotherm

## Abstract

The study investigated the effectiveness of selenium binding from its salt solution by *Candida utilis* ATCC 9950 yeast biomass cultured on a medium prepared from the agro-food industry wastes, containing an available source of carbon and nitrogen. Selenium binding by *C. utilis* yeast strain after 48 h of culturing at 28 °C from aqueous solutions with the addition of 30 mg Se/L reached a value of 2.28 mg Se/g of yeast biomass. The kinetics of selenium binding by the yeasts showed a better fit for the pseudo-second-order kinetic model compared to the pseudo-first-order one. Accumulation stability data were analyzed using the Freundlich and Langmuir isotherm models. The presence of competing anions such as ${\text{SO}}_{4}^{{2 - }}$, and ${\text{HPO}}_{4}^{{2 - }}$ at a concentration of 0.5 mM resulted in about 35% reduction of selenium binding by the examined *C. utilis* strain. FTIR analysis showed that sulfur compounds were involved in selenium biosorption by the yeast. Compounds containing ammonium groups appeared to be very important for selenium binding. The results of the study demonstrated that the yeast can be used to effectively bind selenium from aqueous solution. At the same time, it gives the opportunity to obtain a biomass rich in this deficient element, which can also be used in dietary supplement production.

## Introduction

Selenium is a trace element exhibiting chemical and physical properties intermediate between metals and non-metals due to its position in the periodic table. It is a bioelement essential for the proper functioning of living organisms (Kieliszek and Błażejak 2013). Its required concentration has a very narrow margin between the optimal and potentially toxic dose for humans and animals (Yang et al. 2017; Kieliszek et al. 2016a). The daily intake of this element varies from 10 to 300 µg (Kieliszek et al. 2016b), and exceeding the upper limit can lead to serious health disorders (selenosis). Toxicity of selenium is also dependent on the chemical form in which it occurs. This especially affects absorption of this element by organisms and its bioavailability (Dolgova et al. 2016).

Contamination of ground and surface water with selenium may result from the occurrence of natural geogenic processes or anthropogenic activities, such as oxidation of sulfide minerals (Staicu et al. 2015). In water systems, selenium is found in the form of selenite (${\text{SeO}}_{3}^{{2 - }}$) Se(IV), selenate (${\text{SeO}}_{4}^{{2 - }}$) Se(VI), bi-selenite (${\text{HSeO}}_{3}^{ - }$), and selenous acid (${{\text{H}}_2}{\text{Se}}{{\text{O}}_3}$). The forms commonly found in the pH range of 3.5–9.0 are selenite and bi-selenite (Rajamohan and Rajasimman 2015). An accumulation of these selenium oxyanions in soils, aquifers, and drinking water can threaten the health of animals and humans, and adversely affect the development of plants. The content of selenium varies from several to several 1000 µg/L depending on the type of sewage (Espinosa-Ortiz et al. 2015). Such water is characterized by a bitter taste. To minimize the selenium impact on health, the World Health Organization (WHO) and the Office of Environmental Health Hazard Assessment (OEHHA) recommend that the maximum selenium concentration in drinking water should be below 10 µg/L (Herrero Latorre et al. 2013; Kieliszek and Błażejak 2016).

Yeasts are widely used in the biotechnology and pharmaceutical industry. Large-scale production of yeast biomass has many advantages: (a) it can be obtained using uncomplicated culture techniques, and (b) it can be obtained by culturing on various waste materials from many industries (Khakpour et al. 2014). Moreover, selected yeast species have the GRAS (generally recognized as safe) status, so they are safe for human and animal consumption. In summary, it can be concluded that yeast biomass finds practical use in the utilization of wastes containing metal ions. The use of many wastes and by-products generated by the industry (as components of the culture medium) may lead to the production of wholesome yeast products, examples of which may include protein hydrolysates, B vitamins, enzymes, β-glucans, lipids, and carotenoids (Gientka et al. 2017; Kieliszek et al. 2017; Kot et al. 2016).

Yeasts are characterized by a high degree of interaction with the external environment. The consequence of such interactions is an efficient collection of many elements from the culture environment and their transformation in cellular structures. Yeast bind metals through both chemisorption and bioaccumulation processes. Chemisorption uses the ability of biological materials to collect heavy metals from wastewater by metabolic or physicochemical absorption (Gupta and Rastogi 2008). Physicochemical chemisorption is a process during which metal ions are adsorbed on the surface of the wall–membrane complex. This is the first stage of the element’s binding. The second stage can only take place with the participation of live microorganisms and involves the transport of metal ions to the cellular cytosol (Kieliszek et al. 2015). Numerous elements, including ones essential for life: selenium, zinc, copper, and iron, naturally accumulated by the yeasts. They demonstrate the ability to accumulate large amounts of selenium in their structures (up to 3000 µg/g), which makes it a valuable source of this element in food (Kieliszek et al. 2017). The use of supplements containing organic selenium of yeast origin for deficiency shows a multidirectional beneficial effect on human and animal health (Rayman 2004). At the same time, it should be emphasized that the fundamental source of this element for the human body is always a suitable and balanced diet.

The aim of the study was to determine the characteristics of selenium binding from aqueous solutions by *C. utilis* ATCC 9950 yeast biomass obtained after culturing in media prepared from waste raw materials as available sources of carbon and nitrogen. The effect of initial pH, selenium concentration, the presence of other ions, and temperature on binding efficiency of this element by yeast was determined. Biosorption and kinetic models were also developed to determine the selenium accumulation rate constants.

## Materials and experience

### Microorganism and culture conditions

The material used for the study was *Candida utilis* ATCC 9950 yeast strain from the Museum of Pure Cultures of the Department of Biotechnology and Food Microbiology at SGGW (Poland). The slopes were stored at 4 °C on solid YPD medium.

### Culture media

The yeast strain used for the study was stored in two control media: YPD (BTL, Poland) containing 20 g/L glucose, 20 g/L peptone, and 10 g/L yeast extract, and the medium containing potato wastewater enriched with glycerin (Avantor Performance Materials, Poland) at a concentration of 5% (w/v). The pH value in all media was established at 5.0.

The potato wastewater was obtained during starch production at the PEPEES SA plant in Łomża (Poland). Potato wastewater was subjected to acid–thermal coagulation to partially precipitate the proteins contained in it. The wastewater was acidified with hydrochloric acid to pH 5 and heated at 117 °C for 10 min. The protein was separated from the solution by filtration. Thus, obtained potato wastewater was sterilized (121 °C, 20 min) and stored at room temperature until use. The dry matter content of the obtained potato wastewater extract was 3.21 g/100 mL. It contained about 0.28 g of directly reducing substances and 1.2 g of total protein, constituting a potential source of carbon and nitrogen for the yeast.

### Preparatory and breeding methods

#### Preparation of aqueous solutions containing selenium

The working solution of selenium was prepared by dissolving 0.219 g Na_2_SeO_3_ (Merck, Poland) in 100 mL deionized water, the final concentration of Se(IV) was 1000 mg/L. Experimental aqueous solutions of selenium were prepared in such a way that the final content of this element in 100 mL ranged from 10 to 40 mg Se(IV)/L. The active acidity of aqueous solution of selenium was 5.0. All reagents were sterilized at 121 °C for 20 min.

#### Preparation of inoculum and production media

The yeast inoculum was prepared by inoculating a liquid medium containing potato wastewater and 5% glycerol with a 24-h culture of *C. utilis* yeast strain taken from the slope with a loop. The cultures were incubated on a reciprocating shaker (SM-30 Control E. Büchler, Germany), with a vibration amplitude of 200 cycles/min for 24 h, at a temperature of + 28 °C.

Potato wastewater with 5% glycerol was again used for yeast biomass proliferation, using 10% vol. cell suspensions proliferated in inoculum culture (6.0–8.0 × 10^8^ cfu/mL). The cultures incubated on a reciprocating shaker (SM-30 Control E. Büchler, Germany) at a vibration amplitude of 200 cycles/min at + 28 °C for 24 h.

#### Preparation of yeast biomass for selenium binding from aqueous solutions

**Fig. 1:**
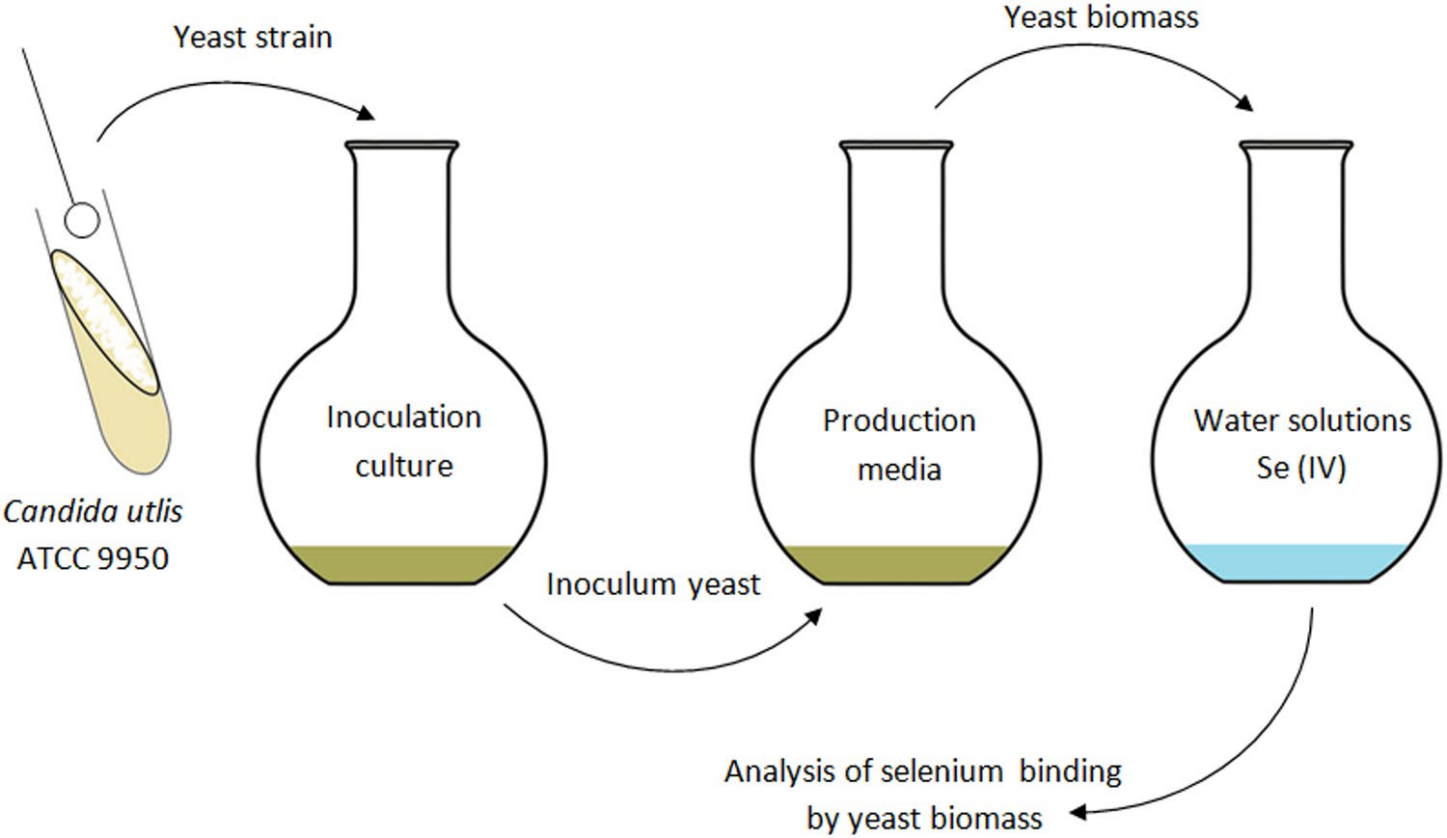
Diagram of culturing and binding selenium from aqueous solutions by *C. utilis* ATCC 9950 yeast

The yeast biomass was obtained by centrifugation (3000×*g*, 10 min, + 4 °C, Centrifuge 5804R Eppendorf, Germany) from proliferation cultures in a medium with glycerol and potato wastewater. Yeast biomass was rinsed twice with sterile distilled water in an amount of 10 g wet biomass/L (with a known content of dry substance 2.5 ± 0.2 g_d.w_) was used to inoculate aqueous control and experimental solutions enriched with selenium (10–40 mg/L). Figure 1 shows the process of preparation, culture of yeast cells and binding of selenium by them. Binding of selenium from aqueous solutions was performed for 48 h at a vibration amplitude of 200 cycles/min at + 28 °C. After the culture process, the yeast biomass was filtered and then centrifuged (3000×*g*, 10 min, + 4 °C, Centrifuge 5804R Eppendorf, Germany). The obtained biomass was lyophilized and stored for further analysis.

#### Determination of selenium binding from aqueous solutions of this element

Selenium content was determined by spectrophotometric method using the Variamine Blue reagent (Kieliszek et al. 2016a). To investigate an effect of pH on selenium (IV) binding by the yeasts, the initial pH of the solution ranged from 2.0 to 10.0. The pH value was corrected using 0.1 M HCl and 0.1 M NaOH solutions at the beginning of the experiment. The pH values were measured using a Consort C863 pH-meter. The effect of competing anions on Se(IV) binding by the yeasts was analyzed using the following salts of chemical elements: 0.5 mM NaNO_3_, Na_2_SO_4_, Na_2_HPO_4_, Na_2_CO_3_, and NaCl. The concentration of anions was selected in accordance with the actual concentration of these compounds found in groundwater. To ensure the accuracy and reproducibility of the collected results, all experiments were performed in triplicates and mean values were presented.

### Mathematical modeling

#### Isothermal examinations

The isotherms of selenium (IV) adsorption by the yeast biomass from aqueous solutions enriched with this element were conducted at various time intervals ranging from 3 to 48 h. Adsorption isotherms were tested at initial Se(IV) concentrations ranging from 10 to 40 mg/L. To evaluate the amount of selenium bound per unit mass of yeast, the adsorption efficiency was calculated as follows:

1 $${q_{\text{e}}}=~\frac{{\left( {{C_{\text{0}}}~ - ~{C_{\text{e}}}} \right)}}{m}~\; \times \;V,$$

where *q*_e_ is the amount of metal ions adsorbed in equilibrium state (mg/g_d.w_.), *C*_e_ is the equilibrium concentration of selenium in solution (mg/L), *V* is the volume of the sample solution (L), and *m* is the weight of biosorbent (g).

The concentration of selenium adsorbed by the yeasts was calculated from the difference between the initial (*C*_0_) and equilibrium (*C*_e_) concentrations of this element in the aqueous solution. The percentage biosorption rate was calculated as

2 $${\text{Biosorption~}}\;(\% )=~\frac{{\left( {{C_{\text{0}}}~ - ~{C_{\text{e}}}} \right)}}{{{C_0}}}~\; \times \;100\% .$$

Adsorption of selenium by the yeasts was interpreted using various models. Langmuir and Freundlich isotherm models were used to describe the experimental data. Langmuir describes the adsorption model leading to the formation of an adsorbate monolayer on the outer surface of the adsorbent and assumes continuous adsorption energy, regardless of the degree of coverage. Langmuir isotherm equations:

3 $${q_{\text{e}}}=~{q_{{\text{max}}}}~\frac{{{K_{\text{l}}}~ \times ~{C_{\text{e}}}}}{{1~+~{K_{\text{l}}} \times {C_{\text{e}}}}}~;$$

after being transformed to a straight line, it is expressed by the formula:

4 $$\frac{{{C_{\text{e}}}}}{{{q_{\text{e}}}}}=\frac{1}{{{q_{{\text{max}}}} \times {K_{\text{l}}}}}+\frac{1}{{{q_{{\text{max}}}}}}~\left( {{\text{linear~form}}} \right),$$

where *q*_e_ is the adsorption capacity, mass of adsorbed substance (element) per unit weight of adsorbent (mg/g_d.w_.), *C*_e_ is selenium concentration in solution in equilibrium state (mg/g_d.w_.), *q*_max_ is the maximum adsorption capacity (mg/g_d.w_.), and *K*_*l*_ is the Langmuir constant (L/mg). The values of *q*_max_ and *K*_*l*_ were determined based on the linear dependence of *C*_e_/*q*_e_ on *C*_e_.

The amount of adsorbed substance depends on the maximum adsorption capacity of the monolayer (*K*_l_, Langmuir constant) and the adsorptive affinity of the adsorbate to the adsorbent (*K*_f_, Freundlich constant). The Freundlich isotherm is an empirical model used to describe multilayer adsorption with interactions between the molecules (Aly et al. 2014; Dutta et al. 2015). Freundlich isotherm equations:

5 $${q_{\text{e}}}=~{K_{\text{f}}} \times {\left( {{C_{\text{e}}}} \right)^{\frac{1}{n}}};$$

after being transformed to a straight line, it is expressed by the formula:

6 $$\ln {q_{\text{e}}}=\ln {K_{\text{f}}}+\frac{1}{n}\ln {C_{\text{e}}}~\left( {{\text{linear~form}}} \right),$$

where *K*_f_ is the Freundlich constant that characterizes the maximum adsorption capacity (L/g), and *n* is the Freundlich constant characterizing the maximum adsorption concentration. Both *K*_f_ constant and *n* were calculated based on the directional coefficients and the coefficients of the straight displacements determined for the Ln*q*_e_ dependence on Ln*C*_*e*_.

The predicted nature of *R*_l_ adsorption process (nondimensional value) (7) was calculated using the following formula (Aly et al. 2014):

7 $${R_{\text{l}}}=\frac{1}{{1+\left( {{K_{\text{l}}}+{C_0}} \right)}},$$

where *K*_l_ is the Langmuir constant (L/mg), and *c*_0_ is the initial concentration of selenium in the solution (mg/L).

#### Biosorption kinetics

The pseudo-first-order kinetic equation (PFO) presented by Lagergren (8) was used for the analysis of selenium adsorption kinetics from aqueous solutions by *C. utilis* ATCC 9950 yeasts. The next pseudo-second-order (PSO) model was presented by Ho and McKay to describe the kinetics of divalent ion adsorption by the adsorbent (9) (Espinosa-Ortiz et al. 2016; Li et al. 2013). These two models are used to describe the rate of biosorption process as well as the parameters determining its rate (Table 1).

**Table 1:** Two models of the biosorption process

Model	Equation	Linear form of the equation	
Pseudo-first order	$\frac{{{\text{d}}{q_t}}}{{{\text{d}}t}}=~{k_1}_{~}\left( {{q_{e1}} - {q_t}} \right)$	$\ln \left( {{q_{{\text{e1}}}} - {q_t}} \right)={\text{ln}}\left( {{q_{{\text{e1}}}}} \right) - \frac{{{k_1} \times t}}{{2.303}}$	(8)
Pseudo-second order	$\frac{{{\text{d}}{q_t}}}{{{\text{d}}t}}=~{k_2}~{({q_{{\text{e2}}}} - {q_t})^2}$	$\frac{t}{{{q_t}}}=~\frac{1}{{{k_2} \cdot q_{{{\text{e2}}}}^{2}}}+\frac{1}{{{q_{{\text{e}}2}}}} \times t$	(9)

where *q*_*t*_ is the number of metal ions adsorbed after time *t*(mg/g), *q*_e_ is the number of metal ions adsorbed at equilibrium state (mg/g), and *k*_1_ and *k*_2_ are the reaction rate constants determined from dependence ln(*q*_e_*–q*_t_) (1/h)

The initial adsorption rate *h* (mg/g/h) was also calculated according to the equation ${k_2} \times q_{{{\text{e}}2}}^{2}$, followed by

10 $$\frac{t}{{{q_t}}}=\frac{1}{h}+\frac{t}{{{q_e}}},$$

where *q*_e_ is the amount of selenium adsorbed at equilibrium state (mg/g_d.w_.) and *t* is the reaction time (h).

#### FTIR spectroscopy

FTIR spectroscopy is based on the analysis of electromagnetic radiation absorbed by a sample. The yeast biomass was obtained by centrifugation (3000×*g*, 10 min, + 4 °C, Centrifuge 5804R Eppendorf, Germany) of aqueous solutions with and without selenium addition. The biomass was then lyophilized. Spectroscopic measurements of yeast cell biomass before and after selenium accumulation process from aqueous solutions were made by Fourier-transform infrared spectroscopy method in the wavelength range from 800 to 4000 cm^−1^ using an FTIR spectrometer (Perkin-Elmer Spectrum One) equipped with beam splitter (KBr) and DTGS detector (deuterated triglycine sulfate). A series of absorption bands was obtained as a result of changes in the frequency range of infrared radiation passing through the test sample. Such an effect causes, as a consequence, excitation and thus a change in the dipole moments of related atoms.

#### Microscopic observations

The surface morphology of *C. utilis* ATCC 9950 yeast cells was evaluated using scanning electron microscopy (SEM) type FEI Quanta 200 FEG with an EDS EDAX microanalyzer (Japan). Dried yeast biomass preparations were applied to a graphite sticker placed on an aluminum table. The preparations were then coated with a thin layer of gold under vacuum to increase the electron conduction and improve the quality of the micrographs. Observations were made in the low vacuum mode (LV mode) using the LFD detector, at a 20–30 kV accelerating voltage.

### Statistical analysis

All results obtained in the study were subject to an analysis of variance using Statgraphics Centurion XVII software. Significance of differences between mean values in particular groups was verified by Tukey’s HSD test for significance level *α* = 0.05. The analysis of the sorption process was conducted based on the results obtained (using the Statistica and SigmaPlot 11 software).

## Results and discussion

### FTIR analysis

**Fig. 2:**
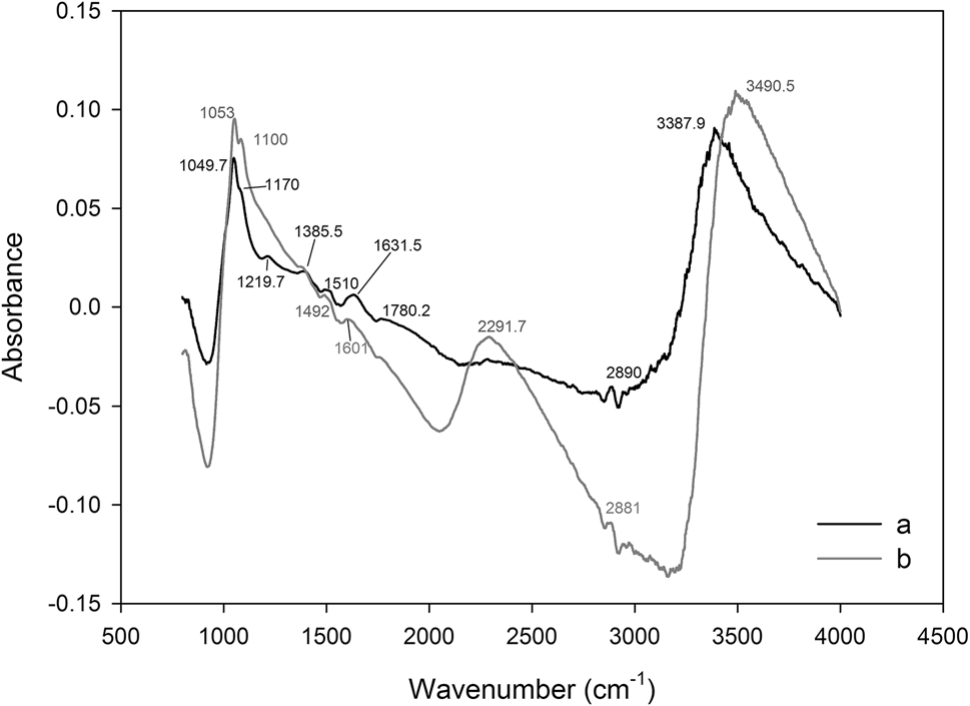
FTIR spectrum of various functional groups before (**a**) and after binding of selenium (**b**) by *C. utilis* ATCC 9950

Fourier-transform infrared spectroscopy (FTIR) is an important tool for functional group identification. It uses the absorption of electromagnetic radiation at specific wavelengths by these groups. Absorption of radiation by molecules is recorded in the form of an absorption spectrum. FTIR spectroscopic analysis was used to obtain information on possible interactions of selenium ions with surface chemical group that this element binds. The results are shown in Fig. 2. The bands at 3387.9–3490 cm^−1^ were due to the presence of limited stretching hydroxyl (–OH) groups in *C. utilis* yeast biomass as well as the presence of stretching vibrations of N–H groups. Strong absorption peak at 2890 cm^−1^ corresponded to alkynes and alkyls (CH_3_ and CH_2_) and the vibrations of C–H stretching groups deformed with amino groups (–NH_2_) decreased to 2881 cm^−1^. Peak complexes at 1631.5 and 1492 cm^−1^ can be attributed to the presence of asymmetric and symmetric stretching vibrations of ionic COO^−^ (carboxylic groups), –NH_2_ (amine), and C–N (amide) groups. The peak at 1510 cm^−1^ corresponding to the N–H bending vibrations was shifted to a lower frequency (1492 cm^−1^) after selenium binding. It can, therefore, be concluded that the amino group may be the main site of this element adsorption by yeast biomass. The signal intensity at 1385.5 cm^−1^ in the control yeast biomass without selenium addition is attributed to phosphate, sulfonyl, and sulfonamide groups (Ramrakhiani et al. 2011). The obtained FTIR results indicate that the adsorption of Se(IV) by yeasts affected chemical groups with sulfur atom in their structure (1380 cm^−1^). It results from similarity in chemical properties of sulfur with selenium, which in consequence could result in possible isomorphic exchange of both elements. Similar results were obtained by Li et al. (2013) investigating selenium accumulation by *Aspergillus* sp. J2 biomass. In their study, the peak at 1382 cm^−1^ (control biomass) became more visible and shifted to 1377 cm^−1^ (biomass with selenium).

The peak of symmetric stretching vibrations of phosphodiester groups was at 1070 cm^−1^. After the process of selenium binding of the biomass, the peak moved to 1100 cm^−1^. Bands near 1049.7 and 1053 cm^−1^ are attributed to the occurrence of stretching vibrations (C–O and O–H), thus showing the presence of hydroxyl groups on the surface of the yeast cell wall. It is a fingerprint that is difficult to interpret; alcohols, polysaccharides, and carboxylic acids are the predominant group of compounds in this vibration band. In addition, this range can also be attributed to the stretching vibration (C–N) band found in protein fractions (Kapoor and Viraraghavan 1997). The peak at 1780.2 cm^−1^ is assigned to the absorption by the carboxylic groups and to the stretching vibrations of conjugated carbonyl groups (C=O). This peak disappeared after selenium adsorption. These phenomena reflect the involvement of carboxylic groups also in selenium binding. After adsorption of selenium by *C. utilis*, a new smaller peak (1601 cm^−1^) and its displacement by 30.5 cm^−1^ were observed in relation to the peak obtained from the control biomass of yeast without selenium addition (1631.5 cm^−1^). The decrease in the number of waves suggests that selenium could interact with the carboxyl and carbonyl functional groups. The above results were confirmed in the study by Nettem and Almusallam (2013), where FTIR analysis of *Ganoderma lucidum* fungus biomass enriched with selenium revealed the presence of amino, carboxyl, hydroxyl, and carbonyl groups. The presented chemical groups were responsible for Se(IV) ion biosorption.

Wide peaks at 2284–2291.7 cm^−1^ indicate the occurrence of stretching vibrations of the amino group. The selenium-enriched biomass of yeast showed the occurrence of this band peak intensity in relation to the control biomass, which could indicate the participation of these chemical groups in element accumulation. The peak obtained from biomass with the addition of selenium at 1219.7 cm^−1^ corresponds to hydroxyl (OH) groups and stretching vibrations of C–N bond. This peak was not observed in the control biomass. Changes in this band indicate that selenium ions are likely to coordinate with nitrogen atoms derived from peptide bonds. Similar results of FTIR analysis were obtained for selenium biosorption by *Cladophora hutchinsiae* algae (Tuzen and Sari 2010). The literature describes many examples demonstrating the great importance of various groups present on the surface of the adsorbent, which are involved in the adsorption process and subsequent accumulation of selenium. Analysis of the FTIR spectrum showed the presence of ionized functional groups (i.e., carboxyl, amino, amide, and hydroxyl) on the surface of *C. utilis* yeast cells. The process of selenium accumulation by yeast cells occurs in the presence of functional groups that exhibit a negative charge on the cell wall surface. An example can be phosphodiester and sulfide bridges (Klis et al. 2002) and negatively charged carboxylic and hydroxyl groups. The presence of proteins, carbohydrates, and lipids on the surface of yeast cells affects the hydrophobicity of their walls and the efficiency of the selenium biosorption process (Kieliszek et al. 2015; Kordialik-Bogacka 2011). In summary, the shift of different absorption bands toward higher or lower frequencies indicates that the chemical interaction of selenium ions with hydrogen atoms of carboxyl, hydroxyl and amine groups was mainly related to the biosorption of this element by ligands of the wall–membrane complex of the biomass of the examined yeast strain.

### SEM characteristics of the yeast

**Fig. 3:**
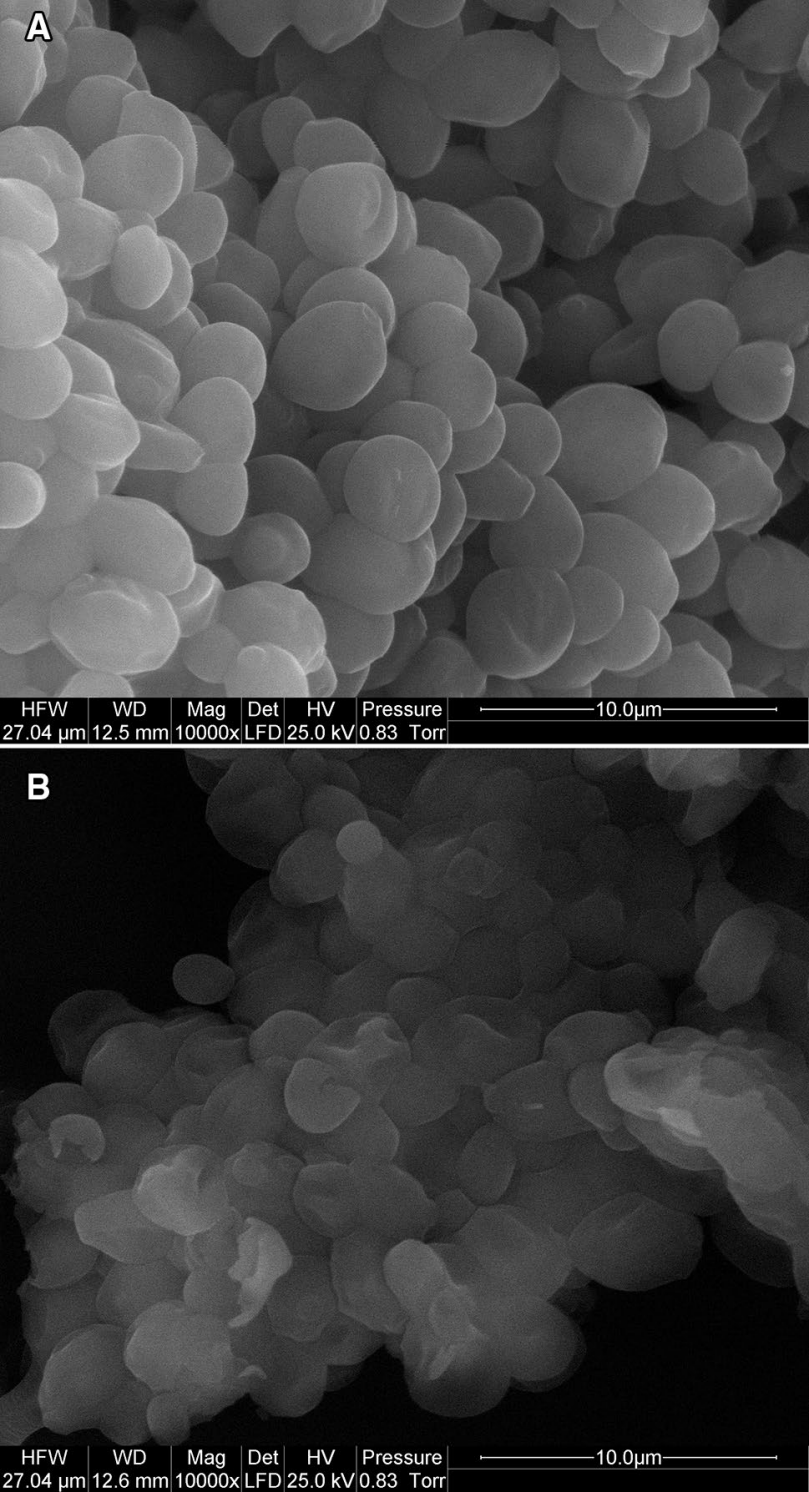
SEM images of *C. utilis* ATCC 9950 cells originating from control aqueous solutions without addition of selenium (**a**) and from media supplemented with 20 mg Se (IV)/L after 24 h incubation (**b**)

The morphology of *C. utilis* yeast cells before and after selenium accumulation process was examined using a scanning electron microscope (SEM). In the image of yeast without the addition of Se(IV) (Fig. 3a), the cells were smooth, oval, cylindrical. They were characterized by an even surface and a constant cell size (2–5 µm). In the case of biomass supplemented with selenium (IV) (Fig. 3b), significant morphological changes were found. The surface of the cells could be covered with selenium. This element could be located on the whole surface of the outer wall–membrane complex of yeast cells as fine particles (10 nm to 1 µm). Yeast cells demonstrated a heterogeneous surface conducive to selenium binding. The cell wall was rough, slightly wavy, with many folds, bends, broken edges, and pores. This appearance of the cells ensured the unveiling of a large number of selenium ion adsorption centers on the surface of the yeast cell wall, increasing the contact surface, which in turn facilitated this binding of this element during the adsorption process. Similar observations were made by Khakpour et al. (2014). *Saccharomyces cerevisiae* PTCC5010 yeast enriched with selenium showed structural porosity.

The main advantages of biosorption technology are its effectiveness in reducing the concentration of nonmetal ions in aqueous solutions to very low levels and the use of inexpensive materials that perform the function of a biosorbent, such as yeast. In conclusion, it can be emphasized that yeast cells characterized by a smooth surface and high porosity (as a sorbent) can be used in the processes of removal of heavy metal ions from aqueous solutions, as well as liquid waste coming from various other branches of industry (Ghorbani et al. 2008).

#### Effect of pH on the biosorption process

**Fig. 4:**
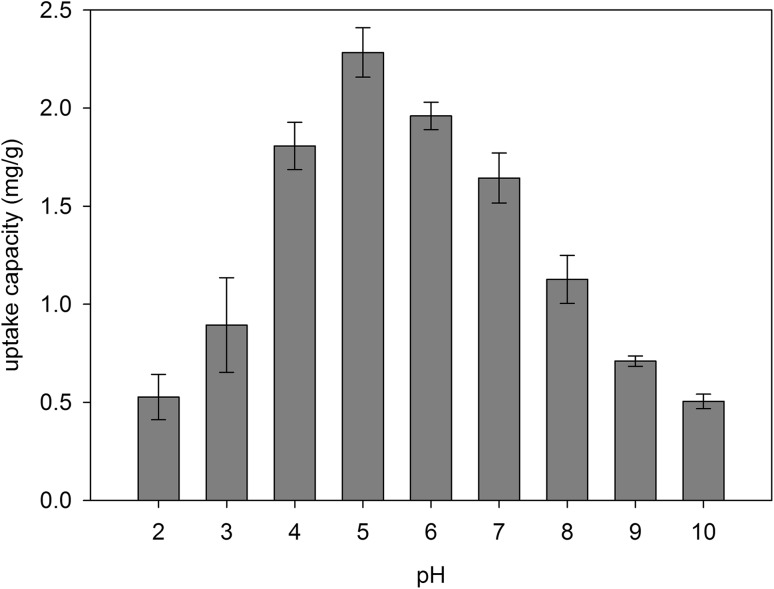
Effect of initial pH on Se(IV) biosorption process by *C. utilis* ATCC 9950 biomass (selenium concentration: 20 mg/L, temp: 28 °C)

The concentration of hydrogen ions (pH) has a very large impact on the biosorption process and subsequent accumulation of various metals from aqueous solutions by yeast. This can be explained based on the surface charge of the adsorbent and the state of the adsorbate in solution. The ionic nature of the solution determines the protonation/deprotonation of the ion-binding sites affecting the availability of functional groups on the surface of the biosorbent, as well as the processes taking place in the solution, for example, reduction, hydrolysis. At pH ranges from 4.0 to 6.0, no significant effect of pH on the Se(IV) biosorption by *C. utilis* yeast was found (Fig. 4). It can be assumed that selenium interactions with ligands of the wall–membrane complex are covalent in nature with some ionic interaction involved (Holmes and Gu 2016). The ability to accumulate this element decreased significantly when the initial pH of the solution increased from 6.0 to 10.0 as well as decreased from 4.0 to 2.0. The amount of selenium bound by yeast was highest at pH 5 (2.28 mg Se^4+^/g_d.w_.) in an aqueous solution supplemented with selenium at a concentration of 30 mg/L. Particularly important for biosorption of this element is the mannoprotein layer, which is the outer protective barrier that determines the permeability of the yeast cell wall. It can be assumed that conditions at pH 5.0 resulted in the exposure of adsorption centers: an increase in the interactive surface of the protein layer, which was associated with an increased exposure of the number of polypeptide chains and amino acid residues (amino group, carboxyl group) in the yeast wall–membrane complex involved in the Se(IV) accumulation process. The minimum efficiency of removal of this element by yeast from aqueous solution was 53 and 48% at pH 2.0 and 10.0, respectively, while the maximum efficiency of selenium removal was maintained at about 77% at pH 6.0. Therefore, pH 5.0 was considered optimal for further experiments. The study presented by Parvathi et al. (2007) showed that amino and carboxyl groups present on the surface of microorganisms are involved in the formation of a characteristic coordination bond with metal ions. Tuzen and Sari et al. (2010) evaluated an effectiveness of Se(IV) biosorption from aqueous solutions using the biomass of *C. hutchinsiae* algae. The highest biosorption of Se(IV) (96%) by green algae was found at pH 5.0, while the lowest (70%) at pH 2.0. A slightly different observation based was made by Li et al. (2013) in their study investigating the effect of pH on Se(IV) binding by *Aspergillus* sp. J2 fungi. The authors noticed the lack of significant pH effect (in the range from 4.0 to 10.7) on the amount of selenium ions adsorbed from the aqueous solution by the fungal biomass. The highest adsorption value (about 4 mg Se^4+^/g_d.w_.) was observed at pH about 5.0. Similar relationships were obtained by Khakpour et al. (2014) for the biomass of *S. cerevisiae* baker’s yeast. The authors showed that the highest level of selenium binding (12.5 mg Se^4+^/g_d.w_.) was observed at pH 5.0.

The yeast cell surface is rich in the presence of many different ligands, such as amine and carboxyl groups, which can promote reactions with positively charged metal ions (Naja and Volesky 2011). According to Brady and Duncan (1994), the carboxyl groups present in cell wall glucans are the major binding sites for divalent metals. With decrease in pH < 4, the group of polypeptide ligands present on the surface of the biomass shows a positive charge. Forming such a barrier consisting of positive charges makes it difficult for selenium ions to access the surface of the biosorbent, which results in a decrease in the sorption and subsequent accumulation of this element in yeast cell structures. The origin of a positive charges can be attributed to inter alia, the presence of basic functional groups and an excessive protonation of the surface (Chojnacka 2010). In the pH range from 4.0 to 6.0, the ionization of carboxyl groups in the amino acid side chains increases the negative charges on the surface of the cell structure. An increase in pH value is accompanied by a decrease in hydrogen ion concentration in the solution (functional groups are deprotonated) with unchanged concentration of selenium ions, and this increases the amount of selenium bound by yeast cells. At higher pH values (7.0–10.0), acid groups (e.g., carboxyl, hydroxyl) and phospholipid phosphates on the surface of the yeast are ionized (Khakpour et al. 2014). The reduction in biosorption process when the pH exceeds 6.0 may be caused by the formation of soluble hydroxyl–metal complexes. In summary, an increase in the percentage of Se(IV) removal from aqueous solution by the biomass of *C. utilis* can be attributed to the ionization of functional groups at pH 5.0. This affects the electrostatic interactions between selenium ions and negative sites on the surface of the yeasts increasing the efficiency of the biosorption process. In addition, the pH of the aqueous solution may also affect the surface charge of the yeasts. This results in the modification of the properties that determine the activity of membrane transporters involved in selenium assimilation by yeast cells.

#### Effect of coexisting ions on the biosorption process

**Fig. 5:**
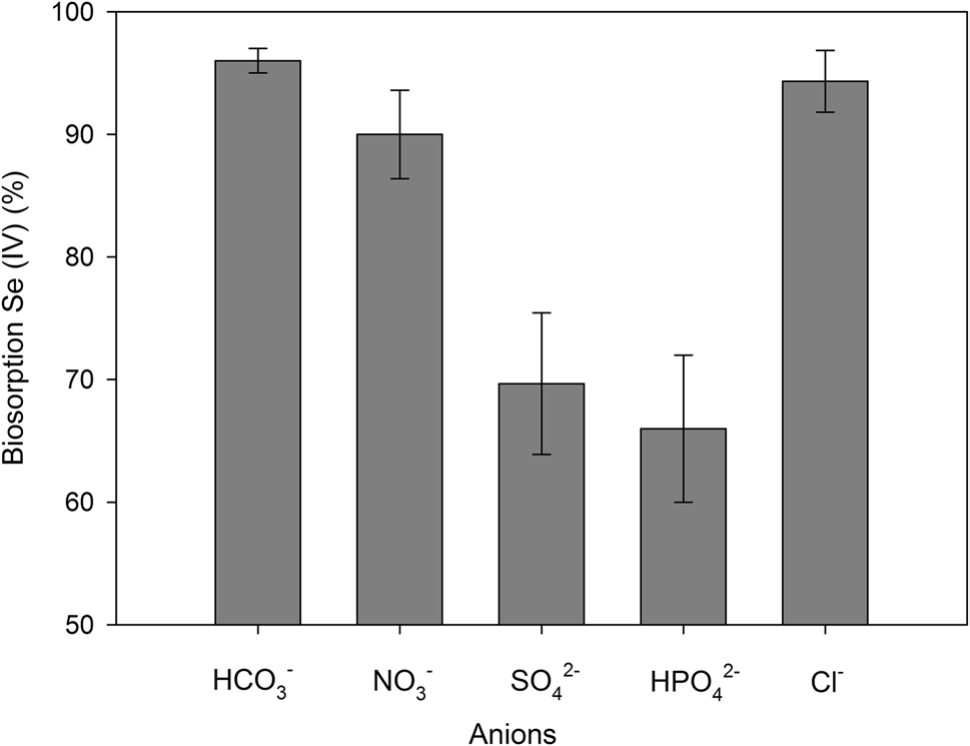
Influence of competing ions on biosorption of Se(IV) by *C. utilis* ATCC 9950 yeast

Anions present in water such as nitrate (${\text{NO}}_{3}^{ - }$), sulfate (${\text{SO}}_{4}^{{2 - }}$), carbonate (${\text{HCO}}_{3}^{{2 - }}$), phosphate (${\text{HPO}}_{4}^{{2 - }}$), and chlorine (Cl^−^) can negatively affect the selenium-binding process by *C. utilis* ATCC 9950 yeasts (Fig. 5). There was no negative influence of carbonate and nitrate in aqueous solutions on the selenium-binding process by *C. utilis* yeast biomass. At a concentration of 0.5 mM, only four of the anions were observed to reduce the biosorption of Se(IV) by yeast compared to aqueous solutions enriched only with selenium. The highest decrease in binding was recorded on using sulfate and phosphate anions, by 31% and 34%, respectively. A similar phenomenon was described in the study by Rajamohan and Rajasimman (2015) where the presence of sulfur and phosphorus oxoanions in the culture medium resulted in the reduction of selenium accumulation by bacteria (active biosorbent) isolated from the bark of the *Eucalyptus camaldulensis* tree. In the light of the literature data, the observed reduction of Se(IV) biosorption among the sulfur anions added can be attributed to similarity in their chemical properties (Vriens et al. 2016). Similar phenomena were described in the study by Li et al. (2013) where sulfur inhibited selenium uptake (by about 1 mg/g) by the biomass of *Aspergillus* sp. J2. The study presented by Gharieb and Gadd (2004) proved that selenium transport by *S. cerevisiae* was inhibited by the presence of essential amino acids containing sulfur (methionine, cysteine, and cysteine) in the culture medium. Similar phenomena were described in the study by Vriens et al. (2016). According to the authors, the presence of sulfur (600 µM) in the medium resulted in the reduction of selenium binding by *Chlamydomonas reinhardtii* by about 55% relative to the control medium where the sulfur content was 150 µM. Confirmation of the results was also obtained from the works of Lazard et al. (2010). The authors demonstrated that the high concentration of phosphates in the medium is associated with reduced adsorption of selenium by yeasts and increased resistance of cells to toxic doses of this element.

In conclusion, the obtained results indicate the possibility of competition of sulfur, phosphate, and selenite ions (IV) for the binding sites on the surface of the yeast cells.

### Kinetics of selenium (IV) biosorption

Biosorption kinetics indicates the rapidity with which substances dissolved in an aqueous solution are adsorbed on the surface of biological materials. This allows to determine the suitability of biological material to be used as a potential selenium biosorbent. Among the many kinetic models described in the literature, those that are based on the order of chemical reactions are most often considered, especially the Lagergren (pseudo-first order), Ho and Mckay (pseudo-second order) models (Espinosa-Ortiz et al. 2016).

**Fig. 6:**
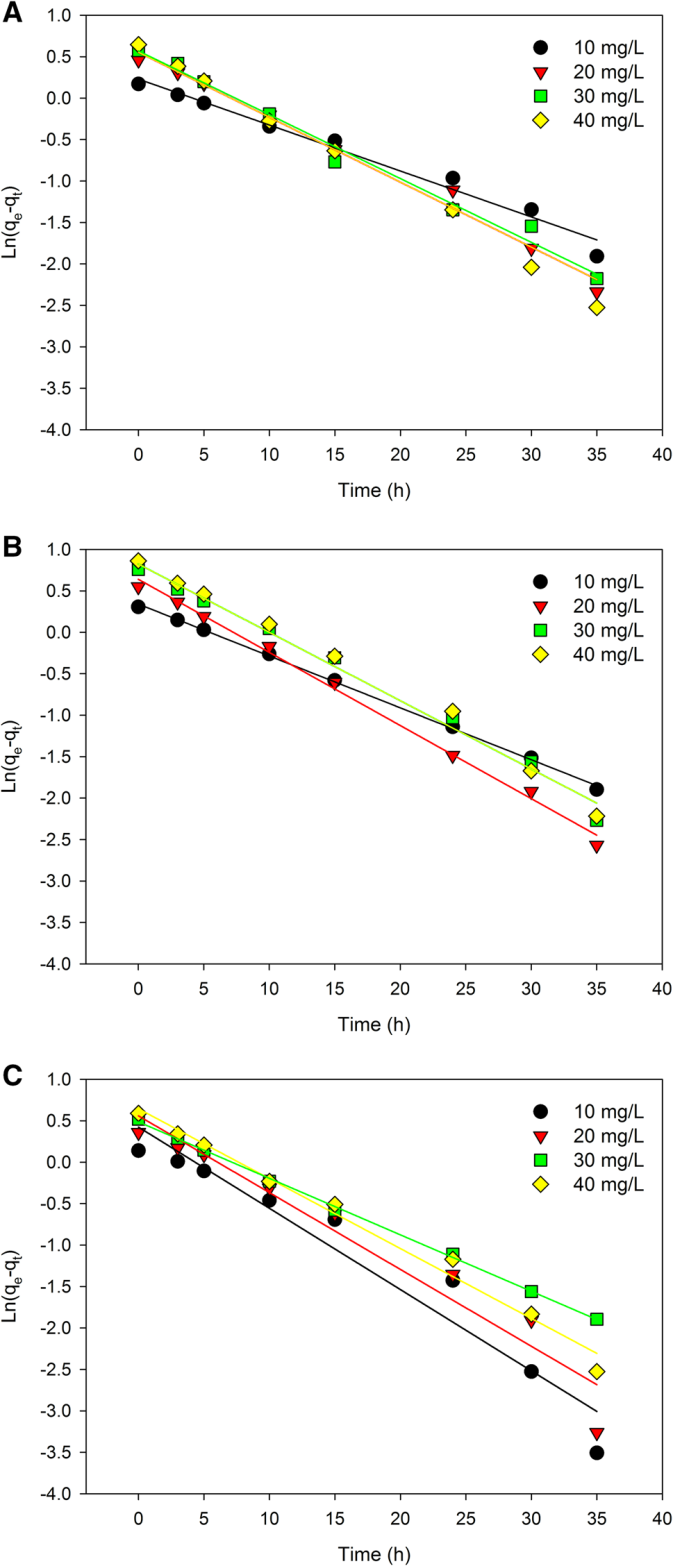
Pseudo-first-order Lagergren for biosorption of selenium by biomass *C. utilis* ATCC 9950 at various temperatures: 22 °C (**a**), 28 °C (**b**), 35 °C (**c**)

Kinetic studies on adsorption of Se(IV) kinetics by yeast cell biomass was performed at temperatures of 22 °C, 28 °C, and 35 °C. Moreover, the amount of selenium adsorbed was monitored in relation to time. Adsorption kinetics usually involves two phases: a stage of rapid removal of the element from aqueous solution, followed by a much slower stage before equilibrium is established. Assuming pseudo-first-order kinetics (Table 2; Fig. 6), the rate of adsorption interactions can be assessed using the simple Lagergren model equation (Sheha and El-Shazly 2010). The biosorption capacity of Se(IV) by *C. utilis* yeast increased with an increasing sorption time, and equilibrium was reached after about 24 h. Thus, the slow biosorption of Se(IV) by the biosorbent could be due to the accumulation of Se(IV) into the yeast cells.

**Table 2:** Kinetic parameters obtained from pseudo-first order and pseudo-second order at different temperatures constants for Se(IV) biosorption by *Candida utilis* ATCC 9950

*C*_0_ (mg/L)	Experimental	Pseudo-first order			Pseudo-second order			
	*q*_e_ (mg/g)	*k*_1_ (1/h)	*q*_e1_ (mg/g)	*R* ^2^	*k*_2_ (g/mg/h)	*q*_e2_ (mg/g)	*h* (mg/g/h)	*R* ^2^
22 °C								
10	1.240	0.055	1.258	0.979	0.019	1.908	0.069	0.989
20	1.663	0.077	1.737	0.989	0.022	2.385	0.125	0.991
30	1.833	0.077	1.766	0.987	0.028	2.420	0.163	0.990
40	1.990	0.089	1.935	0.997	0.038	2.471	0.232	0.998
28 °C								
10	1.420	0.049	1.244	0.962	0.024	2.014	0.097	0.999
20	1.876	0.088	1.896	0.995	0.030	2.452	0.180	0.996
30	2.283	0.082	2.278	0.989	0.027	2.910	0.228	0.997
40	2.530	0.085	2.498	0.992	0.028	3.152	0.278	0.997
35 °C								
10	1.210	0.098	1.527	0.940	0.019	1.986	0.074	0.983
20	1.528	0.092	1.749	0.942	0.027	2.138	0.123	0.990
30	1.793	0.068	1.626	0.998	0.036	2.232	0.179	0.999
40	1.981	0.084	1.904	0.986	0.038	2.438	0.225	0.998

**Fig. 7:**
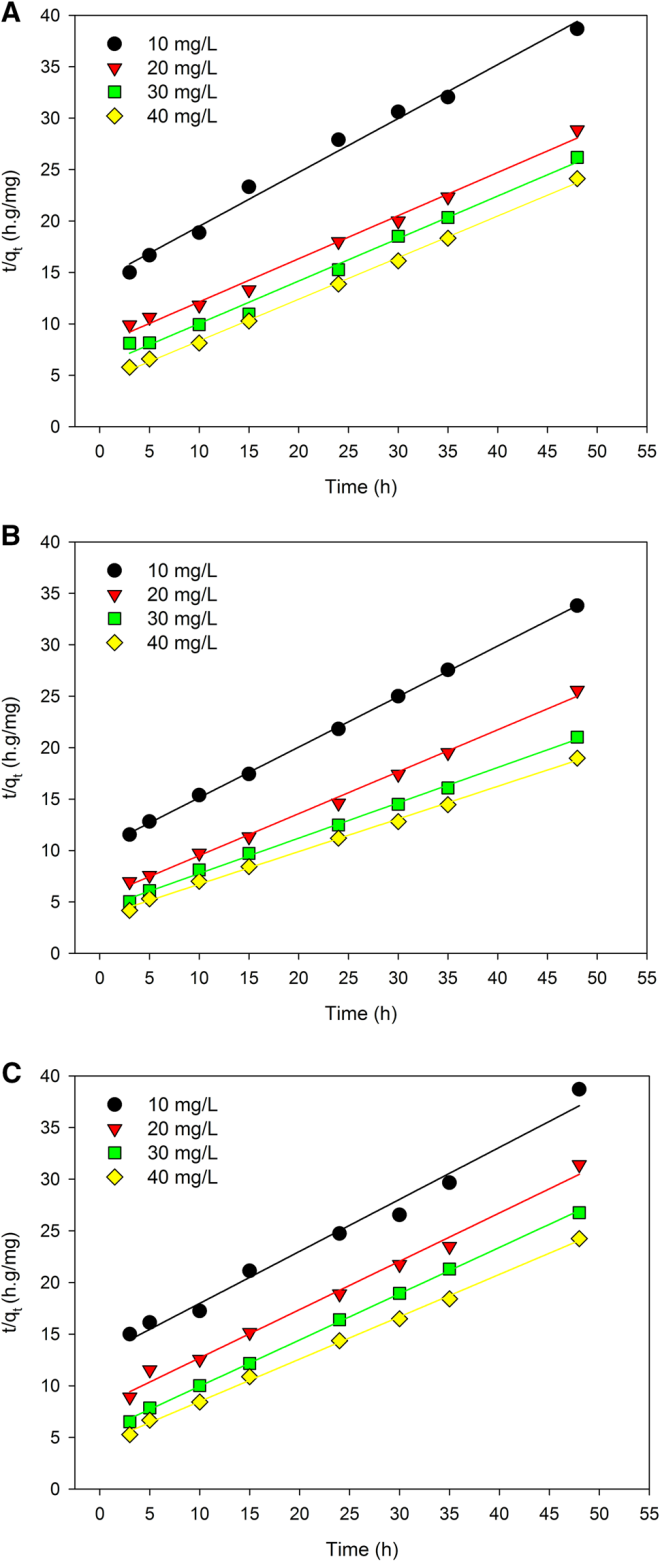
Pseudo-second-order kinetic plots at different temperatures: 22 °C (**a**), 28 °C (**b**), 35 °C (**c**)

Table 2 presents the models for the description of biosorption kinetics. Assuming the correlation coefficient obtained for the linearized forms of pseudo-first and second-order equations as the basic criterion, it can be concluded that selenium adsorption by the biomass of *C. utilis* yeast took place according to the pseudo-second-order model (Fig. 7), hence the conclusion that the adsorption rate could be limited by the speed of the chemisorption process. The rate constant *k*_2_ (mg/g/h) was calculated using the shift coefficient and slope of the straight line determined by the least squares method by plotting *t/q*_*t*_ versus time. The pseudo-second-order kinetics model fits the kinetics of selenium adsorption by yeast biomass since the determined linear regression equations were characterized by a larger square correlation coefficient (close to one) than those obtained for the pseudo-first-order equation, which is demonstrated by *R*^*2*^ values (> 0.99). However, the *q*_*e*_ values obtained from Fig. 7a–c differed from the experimental *q*_*e*_ values by about 20–30%. The rate constant *k*_2_ related to the chemisorption rate increased with an increase in the initial selenium concentration, which indicates the presence of more than one mechanism affecting the selenium binding in the culture by the yeast cell biomass.

Some authors (Rosen and Liu 2009) believe that the transport of this element takes place with the participation of specialized, integral proteins on the surface of the wall–membrane complex or the existence of nonspecific transport of ions complexed with sugar substrates (Kieliszek et al. 2015). The highest value of the rate constant *k*_2_ = 0.038 mg/g/h was found at 22 °C and 35 °C for the selenium concentration of 40 mg/L, while the lowest value of *k*_2_ = 0.019 mg/g/h was obtained at a dose of 10 mg/L under the same conditions of temperature (Table 2). The values of pseudo-second-order sorption rate constants obtained in this study were low (*k*_2_ < 0.05), indicating that the rate of the sorption process was proportional to the number of available binding sites that decreased during prolongation of yeast contact time in aqueous selenium solutions. However, the obtained experimental data again showed better agreement with the pseudo-second-order kinetic model. The diversity of functional groups present in the outer layer of the cell wall (e.g., acyl or carboxylic ones) of *C. utilis* yeast affected the effectiveness of chemical selenium adsorption.

### Isotherms of selenium (IV) sorption

Adsorption of various elements on the surface of yeast cells may be an example of the most effective and the most frequently used method for purifying excess impurities from water.

Analysis of the mathematical description of selenium sorption equilibrium in yeast demonstrated that it is illustrated in the best manner by the Langmuir equation. Langmuir’s theory assumes that sorption occurs at certain homogeneous sites of the adsorbent based on the ability of single-layer sorption (Awual et al. 2015). The efficiency of selenium biosorption by yeast biomass increased with an increasing concentration of this element in aqueous solution. Analyzing the data presented in Table 3, both Langmuir and Freundlich models can describe the experimental data. The values of *R*^*2*^ coefficient for sorption isotherms on *C. utilis* yeast biomass indicated a very good fit of *R*^2^ (> 0.99). At a temperature of 22 °C, a significant difference between the values of the correlation coefficient for both models (Langmuir and Freundlich) was found, that is, 0.999 and 0.983, respectively.

**Table 3:** Langmuir and Freundlich adsorption isotherm model constants for the biosorption of Se(IV) by *Candida utilis* ATCC 9950

Temperature °C	Langmuir				Freundlich		
	*q*_max_ (mg/g)	*K*_l_ (L/mg)	*R*_l_	*R* ^2^	*K*_f_ (mg/g) (L/g)	*n*	*R* ^2^
22	2.411	0.119	0.025 to 0.101	0.999	0.629	3.115	0.983
28	3.345	0.078	0.025 to 0.103	0.991	0.602	2.518	0.997
35	2.471	0.098	0.025 to 0.101	0.993	0.577	2.956	0.998

**Fig. 8:**
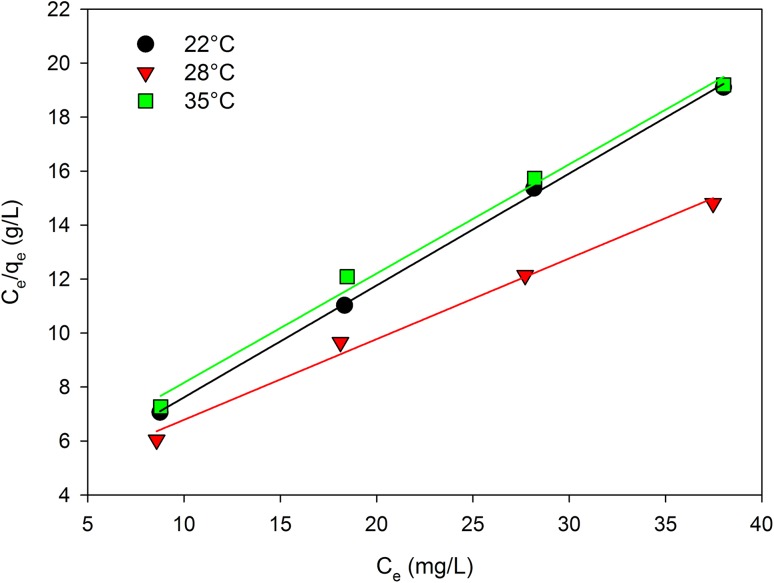
Langmuir isotherm plots for the biosorption of Se(IV) by *C. utilis* ATCC 9950 biomass

The maximum adsorption capacity after 48 h of culture using the Langmuir *q*_max_ model at 22 °C and 28 °C was 2.41 and 3.34 mg/g, respectively. As the temperature increased to 35 °C, no increase in *q*_max_ was observed. The high value of *K*_l_ indicated a higher affinity of yeasts for selenium at 22 °C (0.119) than at 28 °C (0.078) (Fig. 8). The obtained results may indicate the exothermic nature of the selenium adsorption process in yeast.

The results obtained were compared with those published by Li et al. (2013), who showed that *Aspergillus niger* sp. J2 biomass demonstrated Se (IV) biosorption abilities; however, accumulation of selenium increased with an increasing temperature (from 18 to 38 °C), and biosorption better fitted the Langmuir model than the Freundlich model. In the case when *C. hutchinsiae* algal biomass was used in the selenium-binding process, good compatibility with the Langmuir model was found. The biosorption process showed an exothermic character because the selenium-binding capacity decreased with increasing temperature (Tuzen and Sari et al. 2010). The use of an immobilized adsorbent coupled with an organic acid ligand (3-(3-(methoxycarbonyl) benzylidene) hydrazinyl) benzoic acid immobilized on mesoporous silica in selenium binding showed that the Langmuir isotherm model also fitted well with the experimental data (Awual et al. 2015).

To verify the correctness of the Langmuir isotherm application, the distribution coefficient *R*_*l*_ (equilibrium parameter) was calculated. The adsorption process *R*_l_ indicates the course (shape) of isotherms, which may be unfavorable (*R*_l_ > 1), linear (*R*_l_ = 1), preferable (0 < *R*_l_ < 1), or irreversible (*R*_*l*_ = 0) (Aly and Luca 2013). The value of adsorption character (*R*_l_) of selenium by *C. utilis* yeasts can be used to predict whether the sorption system is beneficial both in static and dynamic conditions. The ranges of the equilibrium parameter values obtained at different temperatures and initial concentrations of selenium were positive and below one, which means that the yeast cell biomass acted as a very good selenium adsorbent from aqueous solutions, and consequently, the sorption in the examined conditions was beneficial.

**Fig. 9:**
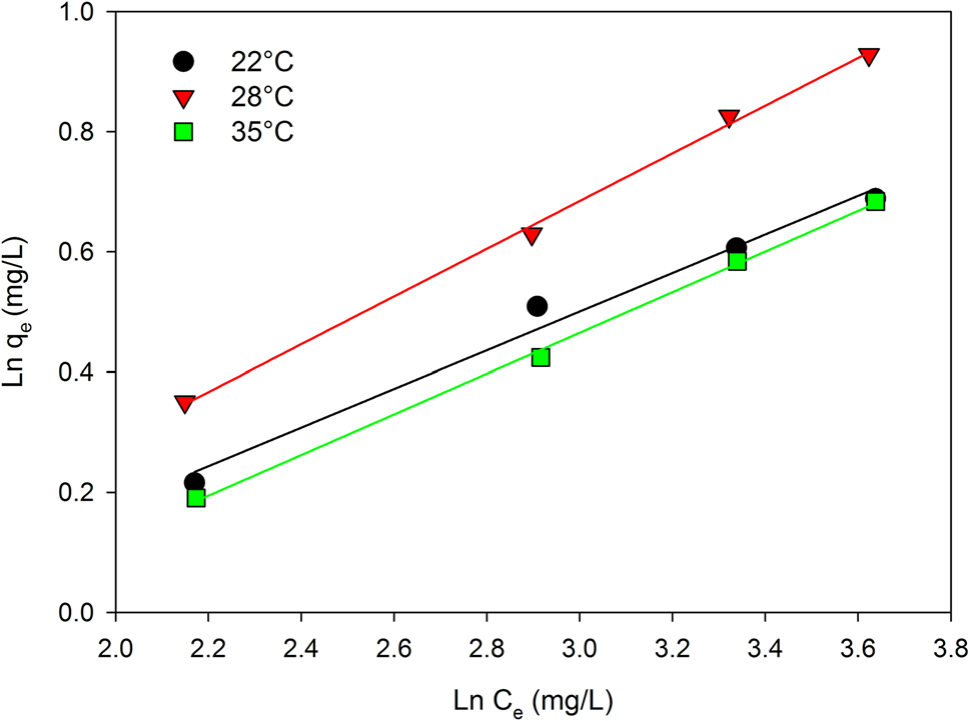
Freundlich isotherm plots for the biosorption of Se(IV) by *C. utilis* ATCC 9950

In the case of the Freundlich model, the constant *n* being a dimensionless parameter makes it possible to evaluate selenium adsorption intensity from aqueous solutions or the inhomogeneity of the sorbent surface (Ahmad et al. 2009). The experimental data were expressed logarithmically in a graphical representation using the linear equation of the Freundlich isotherm (Fig. 9). The numerical values of the model parameters, evaluated at different temperatures together with the correlation coefficient, are given in Table 3. The *n* parameter, which measures the intensity of Se(IV) ion adsorption by yeast, demonstrated values ranging from 2.518 to 3.115. The obtained values of *n* were in the range of 1 < *n* < 10, which confirms that the selenium adsorption process by the yeasts was efficient. The inverse of *n* parameter (1/*n*), which is an irrational fraction, an empirical parameter, gives information about the degree of diversity of sorption sites on the sorbent surface and takes the values 0 < 1/*n* < 1 (Qiu et al. 2018). Based on these, a significant homogeneity of the yeast cell surface can be assumed, since the inverse of the *n* parameter was closer to zero than one. The obtained straight lines indicated that selenium adsorption by *C. utilis* cells fit the examined model.

In summary, adsorption methods provide the opportunity to estimate the practical use of *C. utilis* yeast biomass to remove selenium from aqueous solutions. Moreover, the production and use of yeast cells, in addition to being effective, is also relatively cheap and easy to use on a large scale. Yeast biomass does not generate additional impurities. By choosing the right proportions of yeast biomass depending on the selenium concentration in aqueous solutions, it would be possible to control the properties of such an adsorbent by increasing its adsorption capacity or increasing the rate of adsorbate removal from the solution. Yeast cells obtained from waste substrates (potato wastewater and glycerol) enriched with selenium from various industries could also be used in the production of selenium preparations rich in organic forms of this element, which are well absorbed from the diet by humans and animals, who are deficient in this mineral.

## Conclusion

This study demonstrated that waste products from industry such as potato wastewater and glycerol can be used as ingredients in the culture medium for the production of selenium-enriched yeast. The analysis showed that *C. utilis* ATCC 9950 biomass effectively bound selenium from aqueous solution. The obtained results indicate a significant sorption capacity of yeasts with respect to Se(IV). The data obtained describes well the Langmuir isotherm model, and the adsorption process follows a pseudo-second-order chemical reaction (PSO). The selenium-binding process was dependent on the concentration of hydrogen ions, and the maximum sorption of this element took place at pH5. Among the five anions used, sulfate and phosphate ions significantly interfered with the Se(IV) biosorption. FTIR analysis confirmed the involvement of carboxyl (–COOH), thiol (–SH), amino (–NH), phosphate (${\text{PO}}_{4}^{{3 - }}$), and hydroxyl (–OH) functional groups in the Se(IV) sorption by yeast biomass. In summary, due to easy access as well as low price, the *C. utilis* yeast biomass can be used as an effective adsorbent for Se(IV) removal from aqueous solutions. The experiments were conducted to demonstrate the possibility of *C. utilis* ATCC 9950 yeast biomass application in the production of natural yeast preparations for nutritional purposes using their sorption properties. Large sorption capacity and ease of running the process create the possibility of a new mineral preparation formulation, which can be used in the diet in case of deficiency of this bioelement.
